# Altered endocannabinoidome bioactive lipid levels accompany reduced DNBS-induced colonic inflammation in germ-free mice

**DOI:** 10.1186/s12944-023-01823-1

**Published:** 2023-05-15

**Authors:** Tommaso Venneri, Giada Giorgini, Nadine Leblanc, Nicolas Flamand, Francesca Borrelli, Cristoforo Silvestri, Vincenzo Di Marzo

**Affiliations:** 1 Department of Pharmacy, School of Medicine and Surgery, University of Naples Federico II, Naples, Italy; 2 Joint International Research Unit (JIRU) for Chemical and Biomolecular Research on the Microbiome and its impact on Metabolic Health and Nutrition (MicroMeNu) between Université Laval and the Consiglio Nazionale delle Ricerche (CNR), Institute of Biomolecular Chemistry, Pozzuoli, NA Italy; 3 Centre de recherche de l’Institut universitaire de cardiologie et de pneumologie de Québec, Département de médecine, Faculté de Médecine, Université Laval, Québec, Canada; 4 Centre NUTRISS, École de nutrition, Faculté des sciences de l’agriculture et de l’alimentation (FSAA), Institut sur la Nutrition et les Aliments Fonctionnels, Université Laval, Québec, Canada; 5 Canada Excellence Research Chair on the Microbiome-Endocannabinoidome Axis in Metabolic Health (CERC-MEND), Université Laval, Québec, Canada

**Keywords:** 2,4-dinitrobenzenesulfonic acid (DNBS), Colitis, Endocannabinoids, Microbiome, Antibiotics, Germ-free mice, Inflammation

## Abstract

**Background:**

Gut microbiota are involved in the onset and development of chronic intestinal inflammation. The recently described endocannabinoidome (eCBome), a diverse and complex system of bioactive lipid mediators, has been reported to play a role in various physio-pathological processes such as inflammation, immune responses and energy metabolism. The eCBome and the gut microbiome (miBIome) are closely linked and form the eCBome - miBIome axis, which may be of special relevance to colitis.

**Methods:**

Colitis was induced in conventionally raised (CR), antibiotic-treated (ABX) and germ-free (GF) mice with dinitrobenzene sulfonic acid (DNBS). Inflammation was assessed by Disease Activity Index (DAI) score, body weight change, colon weight-length ratio, myeloperoxidase (MPO) activity and cytokine gene expression. Colonic eCBome lipid mediator concentrations were measured by HPLC-MS /MS.

**Results:**

GF mice showed increased levels of anti-inflammatory eCBome lipids (LEA, OEA, DHEA and 13- HODE-EA) in the healthy state and higher MPO activity. DNBS elicited reduced inflammation in GF mice, having lower colon weight/length ratios and lower expression levels of *Il1b*, *Il6*, *Tnfa* and neutrophil markers compared to one or both of the other DNBS-treated groups. *Il10* expression was also lower and the levels of several *N*-acyl ethanolamines and 13-HODE-EA levels were higher in DNBS-treated GF mice than in CR and ABX mice. The levels of these eCBome lipids negatively correlated with measures of colitis and inflammation.

**Conclusions:**

These results suggest that the depletion of the gut microbiota and subsequent differential development of the gut immune system in GF mice is followed by a compensatory effect on eCBome lipid mediators, which may explain, in part, the observed lower susceptibility of GF mice to develop DNBS-induced colitis.

**Supplementary Information:**

The online version contains supplementary material available at 10.1186/s12944-023-01823-1.

## Summary

Gut microbiota may contribute to colitis development. We show that germ-free mice have blunted responses to DNBS-induced colitis, expressing lower levels of pro-inflammatory cytokines in conjunction with increased levels of anti-inflammatory endocannabinoidome lipids, with marked differences from bacterially-depleted, antibiotic-treated mice.

## Key Messages

What is already known?

Clinical and experimental colitis are associated with gut microbiota dysbiosis and inflammation, both of which are believed to contribute to the development and severity of colitis.

What is new here?

Germ-free mice have decreased inflammation in DNBS-induced colitis in conjunction with increased colonic levels of anti-inflammatory, endocannabinoidome *N*-acylethanolamines as well as 13-hydroxy-octadecadienoicethanolamide (13-HODE-EA) and 13-hydroxy-octadecadienoicglycerol (13-HODE-G). While the biological roles of these HODE compounds have yet to be elucidated, anti-inflammatory roles have been proposed.

How can this study help patient care?

These data suggest that both the gut microbiome and endocannabinoidome bioactive lipids possess therapeutic potential in colitis through their potential immune-modulatory actions.

## Introduction

The human gut (200–300 m^2^ of mucosa) is home to ten trillion different symbionts (50 bacterial phyla and about 1000 bacterial species), collectively known as the gut microbiota [1]. They are acquired at birth and develop in parallel with the host, playing a crucial role in mucosal integrity, nutrient absorption and immune system regulation [2]. The gut microbiome is highly responsive to environmental factors, and its disbalance, known as dysbiosis, is associated with several gastrointestinal diseases, including chronic inflammatory diseases and colorectal cancer [3].

Inflammatory bowel disease (IBD), a term that mainly refers to two diseases, Crohn’s disease (CD) and ulcerative colitis (UC), is a recurrent and lifelong condition characterised by chronic inflammation of the gastrointestinal tract. It represents a global health burden affecting millions of people, with increasing incidence and prevalence worldwide [4]. There are hypotheses of IBD etiopathogenesis, but the most accepted one is an abnormal immune response to antigens in the presence of a predisposing gut microbiota composition [5].

The endocannabinoidome (eCBome), or “expanded endocannabinoid system”, includes, in addition to the cannabinoid CB1 and CB2 receptors, their ligands, anandamide (*N*-arachidonoylethanoamine; AEA) and 2-arachidonoylglycerol (2-AG) and the enzymes responsible for their biosynthesis and degradation, other structurally-related lipid mediators, such as different *N*-acylethanolamines (NAEs) and 2-monoacylglycerols (2-MAGs), as well as lipoaminoacids, such as *N*-acylglycines and *N*-acyltaurines, and *N*-acylated neurotransmitters, such as *N*-acylserotonins [6]. Additionally, some polyunsaturated members of these families of lipids can act as substrates for lipoxygenases involved in arachidonic acid metabolism and participating in inflammation, such as 15-lipoxygenase, which, for example, can catalyse the oxidation of *N*-linoleyl-ethanolamine and 2-linoleoyl-glycerol to the corresponding 13-hydroxy-derivatives, 13-HODE-EA and 13-HODE-G [7, 8]. This complex group of lipid mediators has been shown to bind G protein coupled receptor, such as GPR18, 55, 110 and 119, but also ion channels, such as TRPV1, TRPM8 and TRPA1, as well as PPARs receptors [9]; enzymes for their synthesis and degradation (in this latter case often common with NAEs or 2-MAGs) have also been described for eCBome mediators [7, 10].

Manipulation of the eCBome has been shown to be of great benefit in attenuating inflammation in mouse models of colitis. First of all, pharmacological or genetic modulation of several receptors belonging to the eCBome (TRPV1, TRPA1, PPARα, and GPR55) [11–13], administration of eCBome lipid mediators (PEA, OEA) [14–17] and pharmacological blockade of MAGL, FAAH and NAAA [15, 18, 19], enzymes that metabolise eCBome mediators, showed significant anti-inflammatory effects in mouse models of colitis. While mostly anti-inflammatory per se, AEA and 2-AG can contribute to inflammation also by producing a variety of bioactive lipids with pro-inflammatory action following their hydrolysis [20]. Furthermore, supplementation with omega-3-fatty acids, which reduce the levels of both AEA and 2-AG in several tissues, are able to decrease the severity of experimentally induced colitis [21]. Finally, the eCBome has been shown to be a target of phytocannabinoids extracted from *Cannabis sativa* [10] and several studies have shown the anti-inflammatory effects of these compounds (CBD, CBG, CBDV and CBC) in different models of intestinal inflammation in mice and rats [22–26].

Recently, the existence of an interaction between the eCBome and the gut microbiome (mBIome), known as the eCBome-mBIome axis, has been reported [27, 28]. The gut microbiota produces a plethora of metabolites that influence intestinal homeostasis [2], with some of these molecules being structurally similar to lipid mediators belonging the eCBome and, therefore, it is not a surprise that the eCBome and gut microbiome mutually influence each other, playing a role in several physiological and physio-pathological functions [29].

On this background, the aim of this work was to investigate whether the interaction between the eCBome and the gut microbiome has an impact on the development of IBD using the DNBS colitis model; this aim was achieved by manipulating the gut microbiota with two methods of depletion of gut bacteria (treatment with antibiotics and germ-free mice, which, to the best of our knowledge, have never been compared in the context of DNBS-induced colitis), and by investigating if these alterations were correlated with altered development of colon inflammation and modifications in the eCBome.

## Materials and methods

### Drugs and reagents

2,4-dinitrobenzenesulfonic acid (DNBS), antibiotics cocktail (ampicillin, streptomycin and clindamycin) and the reagents for protein and lipid extraction were purchased from Sigma Aldrich, Canada; all the reagents for mRNA extraction and retrotranscription were purchased from FisherScientific, Canada and Qiagen, Germany. All chemicals and reagents employed in this study were of analytical grade.

### Animals

Conventionally raised (CR) (6 weeks old) and germ-free (GF) (8 weeks old) Balb/c male mice were purchased from Taconic (Taconic Bioscience, NY, USA) and maintained in the animal facility of the Institut Universitaire de Cardiologie et Pneumologie de Québec (IUCPQ, QC, Canada). Mice were housed in single cage under a 12 h:12 h light dark cycle with ad libitum access to NIH-31 Open Formula Autoclavable Diet (Zeigler, PA, USA) and water. GF mice were housed in axenic status and fecal samples as well as litter samples from each cage were tested to ensure that the GF condition was maintained throughout the whole experiment. Both GF and CR mice were acclimatized for at least one week prior to start with the procedures. All mice were fasted for 12 h overnight before the intracolonic injection of DNBS. Mice were randomly allocated in different experimental groups. All the animal procedures were validated and approved by Laval University animal ethics committee (CPAUL, 2020 − 587). Animal studies are reported in compliance with the ARRIVE guidelines [30]. G*Power was used for sample size calculation [31].

### Dinitrobenzenesulfonic acid (DNBS)-induced colitis

DNBS was dissolved in 30% ethanol/ringer lactate solution and administrated into the rectum (120 mg/kg, 100 µL/mouse) by a polyethylene catheter (1 mm in diameter) inserted approximately 3.0 cm proximal to the anus. In preliminary experiments this dose of DNBS was found to induce remarkable colonic damage associated with high reproducibility and low mortality. After 3 days, all mice were euthanized by cardiac puncture during isoflurane anaesthesia, the mice abdomen was opened by a midline incision and the colon removed, isolated from surrounding tissues, length measured, rinsed, weighed and then processed. Mice body weight was measured every day during the treatment period [25, 32]. For biochemical analysis, tissues were snap frozen in liquid nitrogen and kept at − 80 °C until the use (for MPO assay and lipidomic analysis) or stored in RNAlater (for qPCR analysis).

### Antibiotic-induced gut bacteria depletion

To eliminate the gut microflora, mice were treated with a mix of ampicillin (1 mg/mL), streptomycin (1 mg/mL) and clindamycin (1 mg/mL) in their drinking water [33]. After two weeks of antibiotic treatment, mice started the DNBS-induced colitis protocol as described before.

### Evaluation of myeloperoxidase activity

MPO activity, a peroxidase enzyme used to quantify the neutrophil infiltration in whole-tissue colons, was determined as previously described [25, 34]. Full-thickness colons were mechanically homogenized using the Qiagen TissuelyserLT (50 Hz for 30 s, two cycles) in 0.5% w/v hexadecyltrimethylammonium bromide (HTAB) in 10 mM 3-(N-morpholino)-propanesulfonic acid (MOPS), using a ratio of 50 mg tissue/mL MOPS. The homogenates were centrifuged for 20 min at 15,000×g at 4 °C and an aliquot of the supernatant was incubated with sodium phosphate buffer (NaPP pH 5.5) and a 16 mM 3,3′,5,5′-tetramethylbenzidine solution in DMSO. After 5 min, hydrogen peroxide (10 mM in NaPP) was added and the reaction was stopped with acetic acid (2 M). The rate of change in absorbance was measured by a spectrophotometer at 650 nm. MPO activity was expressed as relative amount normalized with the control group.

### Gene expression analysis by quantitative PCR

Total RNA from murine tissues was extracted and purified (Qiacube, Qiagen, RNeasy 96 kit), quantified (Nanodrop, Thermofisher), and retrotranscribed (High-Capacity cDNA Reverse Transcription Kit) using the manufacturer protocols. Quantitative real-time PCR was carried out in CFX Opus 384 Real-Time PCR System (Biorad) by using SYBRGreen detection. Selective primers were purchased from Integrated DNA Technologies (Iowa, USA) (Table 1). Each sample was amplified simultaneously in a triplicate in one-assay run (maximum ΔCt of replicate samples < 0.5), and a non-retro-transcription control (NRC), a non-template control (NTC) as well as a standard curve from consecutive fivefold dilutions (100–0.16 ng) of a cDNA pool representative of all samples was included for PCR efficiency determination. Data normalization was performed by using as a control the Ct from *Hprt*, constitutively expressed protein; differences in mRNA content between groups were calculated as normalized values by use of the 2^−∆∆Ct^ formula.

**Table 1:** List of primers used in RT-PCR analysis

Gene name	Forward	Reverse
Murine *Il1b*	5’-TATACCTGTCCTGTGTAAA-3’	5’-TTGACTTCTATCTTGTTG-3’
Murine *Il10*	5’-TTATTACCTCTGATAATCT-3’	5’-CCATCATATAATATAATCTCC-3’
Murine *Il6*	5’-CCTGGAGTACATGAAGAA-3’	5’-TGGTTGAAGATATGAATTAGAGT-3’
Murine *Tnfa*	5’-GGTGCCTATGTCTCAGCCTCTT-3’	5’-GCCATAGAACTGATCAGAGGGAG-3’
Murine *Cxcl1*	5-CCAAACCGAAGTCATAGCCA-3’	5’-GTGCCATCAGAGCAGTCT-3’
Murine *Elane*	5’-GTGAACGTATGCACTCTGGT-3’	5’-CTCGGATGAAGGAGTCAATGC-3’
Murine *Ly6g*	5’-GCGTTGCTCTGGAGATAGAAG-3’	5’-TTGACAGCATTACCAGTGATCT-3’
Murine *Arg1*	5’-GAATGGAAGAGTCAGTGTGGT-3’	5’AGTGTTGATGTCAGTGTGAGC-3’
Murine Hprt	5’-TTGACACTGGTAAAACAATGC-3’	5’-GCCTGTATCCAACACTTCG-3’

### HPLC-MS/MS analysis for quantification of lipid mediators

Lipid (namely NAEs, 2-MAGs and 15-lipoxygenase NAE/2-MAG derivatives) extraction was performed following the Bligh and Dyer method [35]. Briefly, approximately 10 mg of colon tissues were homogenized with a using a tissue grinder then suspended in 0.5 mL of Tris-HCl (50 mM pH 7). 0.5 ml methanol containing 5 ng of deuterated standards and acetic acid (0.5%) was next added to the mixture. An organic phase extraction with chloroform was performed on each sample by adding 1 mL of chloroform in the mixture, vortexing for 30 s and centrifuging at 4000 g for 5 min. This was repeated three times for a total of 3 mL of chloroform. The organic phases were then collected and evaporated under reduced pressure using a speedvac evaporator dissolved in 50 µL of mobile phase containing 50% of solvent A (water + 1mM ammonium acetate + 0,05% acetic acid) and 50% of solvent B (acetonitrile/water 95/5 + 1 mM ammonium acetate + 0.05% acetic acid). After that, 40 µL of each sample were injected into an HPLC column (Kinetex C8, 150 × 2.1 mm, 2.6 μm, Phenomenex) and eluted at a flow rate of 400 µL/min using a discontinuous gradient of solvent A and solvent B. Quantification of lipid mediators was carried out by using an HPLC system interfaced with the electrospray source of a Shimadzu 8050 triple quadrupole mass spectrometer and using multiple reaction monitoring in positive ion mode the compounds of interest and their deuterated homologs [28].

### Statistical analysis

Statistical analysis was performed using GraphPad Prism version 8.1.2. Data are expressed as the mean ± SEM of n experiments. Outliers were identified by ROUT test. Data were compared using two-way ANOVA followed by Fisher’s Least Significant Difference (LSD) test. Correlation between inflammatory parameters, genes and eCBome mediators measured was performed using Pearson’s r correlation coefficient with a two-tailed post-test to assess statistical significance. Data were expressed using a heat map matrix. p < 0.05 was considered to be significant.

## Results

### Gut microbiota impairment in GF, but not ABX, mice affects some macroscopic inflammatory parameters in DNBS-induced colitis

**Fig. 1:**
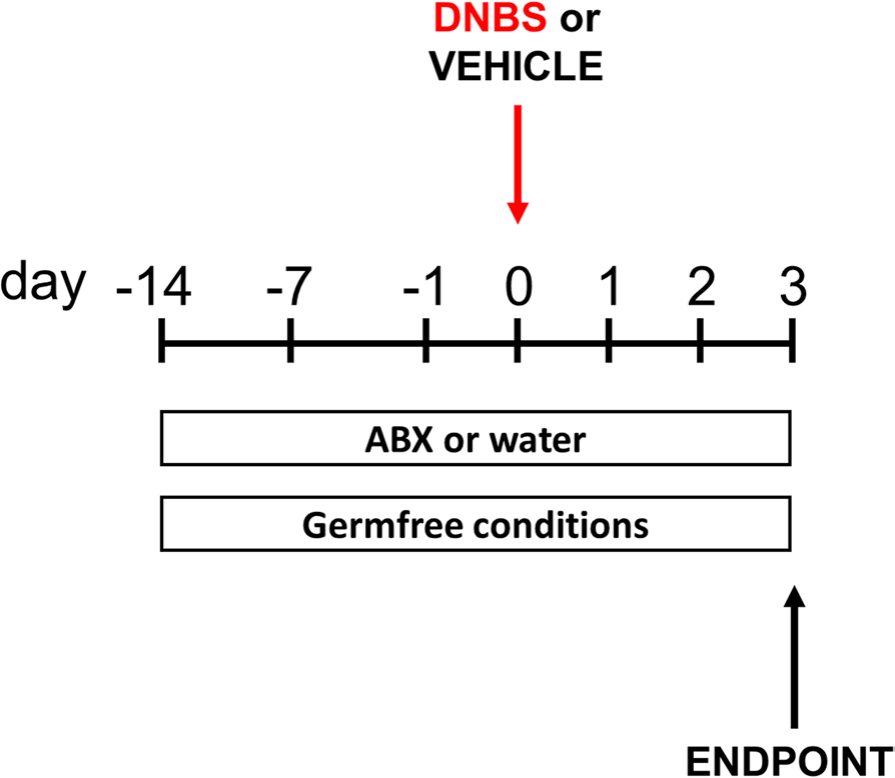
Schematic representation of experimental procedures used to alter gut microbiota composition (antibiotic pre-treatment [ABX] or germ-free conditions) in the DNBS-induced model of ulcerative colitis. An antibiotic cocktail containing ampicillin (1 mg/mL), streptomycin (1 mg/mL) and clindamycin (1 mg/mL) was given to mice for a fortnight prior to DNBS administration and until the animals were killed. DNBS was rectally injected into the mouse colon at the dose of 120 mg/kg; mice were killed three days after DNBS administration

To understand the role of the eCBome – mBIome axis in the development of experimental colitis, we used two animal models which induce a depletion of the gut microbiota: mice treated with an antibiotic cocktail (ABX), and mice raised under germ-free (GF) conditions (Fig. 1). The effects of DNBS on ABX and GF mice were compared with those on conventionally-raised (CR) mice. Importantly, we attempted to quantify the effect of the antibiotic cocktail in ABX mice, and the effect of DNBS treatment on this by 16 S rDNA sequencing. However, we were unable to generate a significant number of sequencing reads in these mice (data not shown), indicating that the cocktail efficiently suppressed the number of bacteria within the gut.

**Fig. 2:**
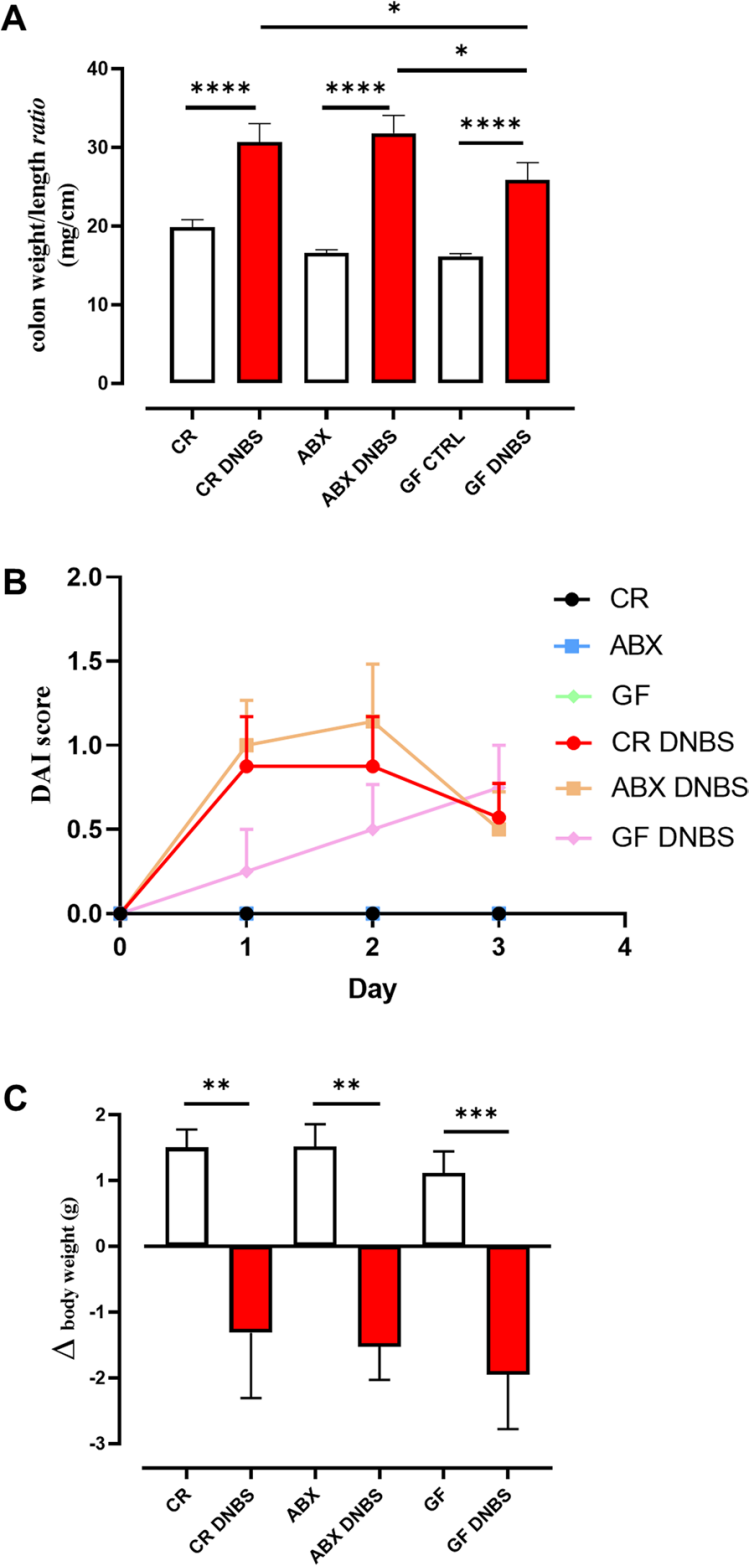
Effect of DNBS administration in conventionally raised (CR), antibiotic-treated (ABX) and germ-free (GF) mice on macroscopic inflammatory parameters. (**A**) colon weight/colon length ratio, (**B**) disease activity index (DAI) score and (**C**) body weight loss. DNBS was rectally injected into the mouse colon at the dose of 120 mg/kg; ABX mice received an antibiotic cocktail (ampicillin, streptomycin and clindamycin 1 mg/mL each) starting 14 days before DNBS administration and for the whole length of the experiment. Mice were killed three days after DNBS administration. Data are expressed as mean ± SEM of 6–8 mice for each experimental group. Data were statistically analyzed using two-way ANOVA followed by the Fisher’s LSD test. *p < 0.05, **p < 0.001, ***p < 0.001 and ****p < 0.0001. The DAI score of the CR, ABX and GF CTRL groups was always equal to 0

Intracolonic administration of DNBS elicited remarkable inflammation in the mice regardless of the status of the mBIome (Fig. 2). As shown by the colon weight/length ratio, a macroscopic parameter of colon inflammation, CR, ABX and GF mice treated with DNBS showed a statistically significant increase in this parameter compared to their own controls; interestingly, the ratio was significantly lower (suggesting potentially lower inflammation) in GF mice treated with DNBS than DNBS-treated CR and ABX mice (Fig. 2A). Accordingly, when analyzing the DAI score, DNBS-treated CR and ABX mice showed a sudden increase in this parameter on day 1 and 2 and a slight decrease on day 3 (Fig. 2B); in contrast, the DAI score in DNBS-treated GF mice was lower in the first days of the protocol, although not statistically significant, and reached the same value as the other two groups only on the last day (day 3), suggesting that DNBS-induced colitis may develop with different kinetics in GF mice. Despite this, however, all DNBS-treated mice, regardless of their microbiota status, showed body weight loss of the same intensity when compared to control mice (Fig. 2C).

### Gut microbiota impairment alters the expression of cytokines and MPO activity in healthy and DNBS-treated mice

**Fig. 3:**
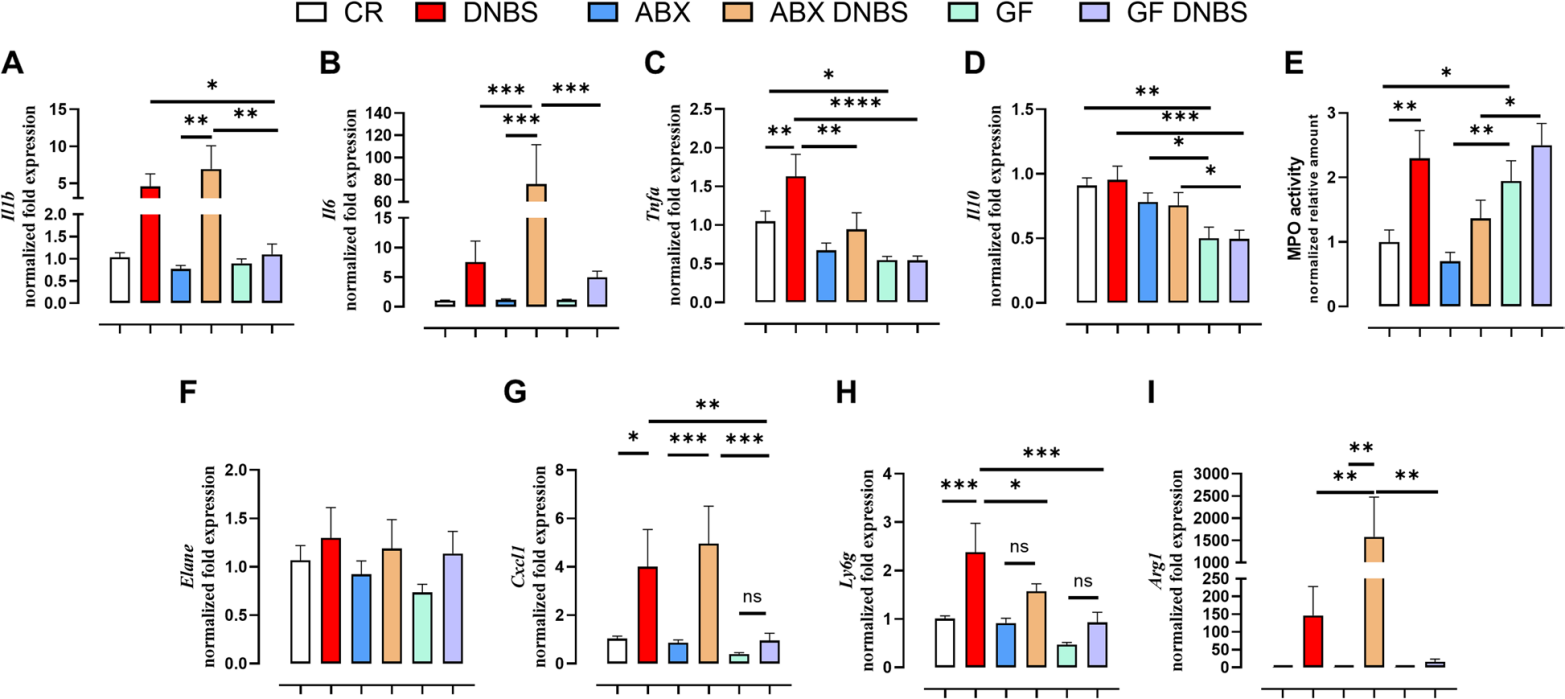
Inflammatory parameter gene expressions in colon tissue from conventionally raised (CR), antibiotic- treated (ABX) and germ-free (GF) mice in healthy condition or under DNBS-induced inflammation. (**A-D**) expression levels of cytokines involved in inflammation, **E**) MPO activity (expressed as normalized relative amount), **F-I**) expression levels of neutrophil markers DNBS was rectally injected into the mouse colon at the dose of 120 mg/kg; ABX mice received an antibiotic cocktail (ampicillin, streptomycin and clindamycin 1 mg/mL each) starting 14 days before DNBS administration and for the whole length of the experiment. Mice were killed three days after DNBS administration. Data are expressed as mean ± SEM of 6–8 mice for each experimental group. Data were statistically analyzed using two-way ANOVA followed by the Fisher’s LSD test. *p < 0.05, **p < 0.01 and ***p < 0.001

To take a closer look at the molecular pathways of inflammation, we examined the gene expression levels of a number of cytokines (*Il1b*, *Il6*, *Il10* and *Tnfa*) and MPO activity (a marker of neutrophil infiltration) (Fig. 3). All mice, regardless of microbiota status, showed similar basal levels of *Il1b* and *Il6* expression. Treatment with DNBS resulted in an increase in the expression of *Il1b* and *Il6* in CR and ABX mice, this effect being, however, significant only in ABX mice (*Il1b*: p value for CR vs. CR DNBS 0.0526; *Il6*: p value for CR vs. CR DNBS 0.720). Furthermore, while DNBS induced a similar level of expression of *Il1b* in ABX mice as compared to CR mice, it induced significantly higher levels of *Il6* expression. No changes in *Il1b* and *Il6* expression were observed in GF mice (Fig. 3A and B). CR mice showed basal *Tnfa* expression levels that were significantly higher than GF mice. DNBS treatment increased *Tnfa* expression levels in CR and ABX mice (although the latter was not statistically significant), but not in GF mice (Fig. 3C). GF mice showed lower basal expression levels of *Il10* than CR and ABX mice; in all mice, *Il10* expression levels were unaffected by DNBS administration (Fig. 3D). Finally, basal MPO activity was significantly higher in GF mice than in CR and ABX mice. DNBS administration resulted in a significant increase in MPO activity in CR, but not in ABX and GF mice (Fig. 3E). These data suggest that neutrophil activity/levels may be altered within the colons of ABX and GF mice in response to DNBS. To test this, we went on to measure the expression of several neutrophil activation and recruitment markers. While DNBS resulted in weak trends towards increased neutrophil elastase (*Elane*) expression, these effects were all statistically insignificant (Fig. 3F). In contrast the expression of the chemokine *Cxcl1*, which is involved in neutrophil recruitment and activation [36, 37] was strongly upregulated in response to DNBS in CR and ABX mice, but not in GF mice (Fig. 3G). *Ly6g*, a neutrophil-expressed maturation marker [38, 39] was significantly increased by DNBS in CR mice, but only showed trends towards increases in ABX and GF mice (Fig. 3H), while the activated neutrophil marker Arginase (*Arg1*), the release of which is associated with T-cell dysfunction (which is implicated in the development of inflammation in IBD [40, 41], was massively upregulated in CR and ABX mice in response to DNBS (though only significantly in the latter), with relatively no effect in GF mice (Fig. 3I). All these data suggest that mice treated with antibiotics and, particularly, those raised under germ-free conditions show a different evolution of the DNBS-induced inflammatory response as compared to CR mice.

### Conventionally raised and germ-free mice show different levels of eCBome mediators under healthy and inflammatory conditions

**Fig. 4:**
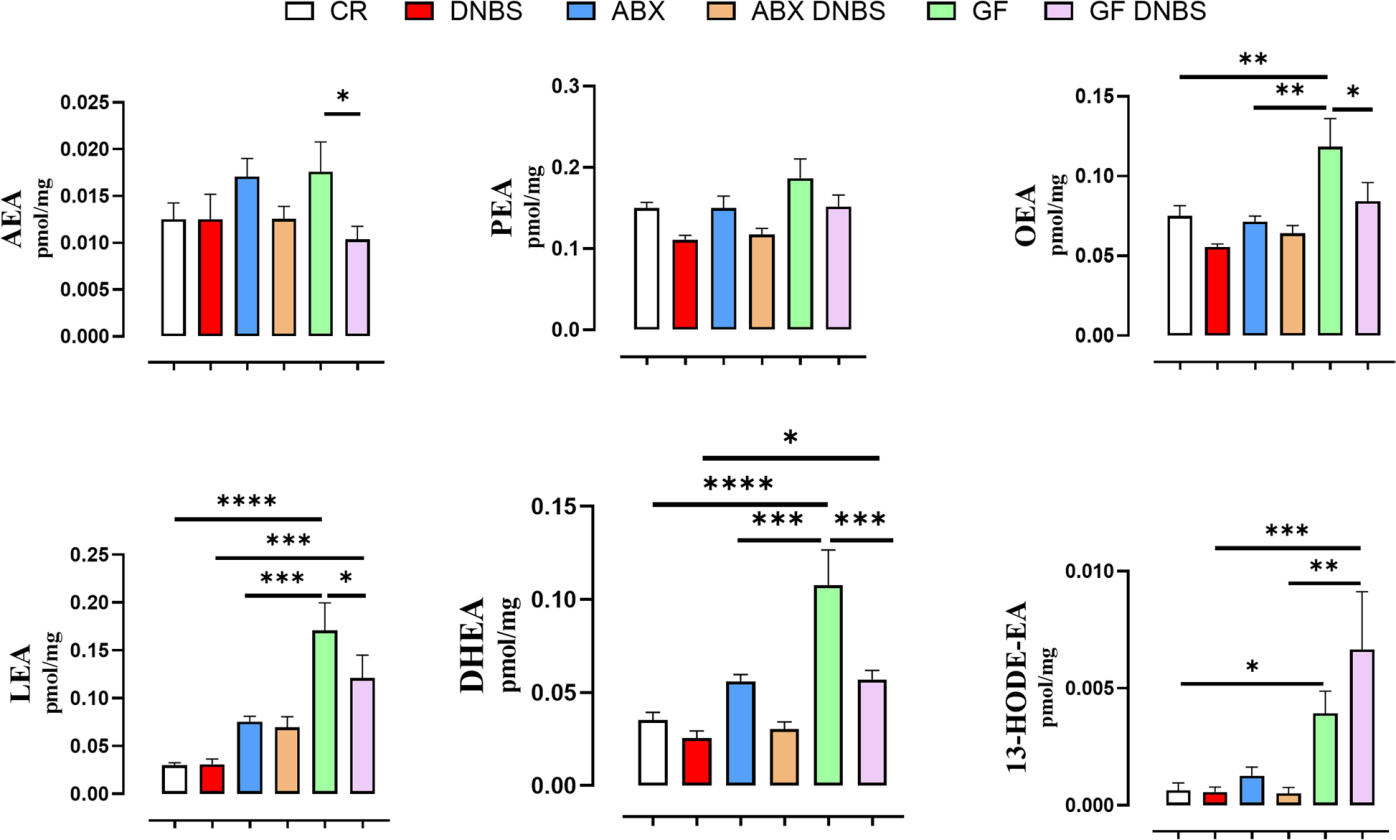
NAE, and 12-lipoxygenase NAE derivative, levels in colon tissue from conventionally raised (CR), antibiotic- treated (ABX) and germ-free (GF) mice in healthy condition or under DNBS-induced inflammation. DNBS was rectally injected into the mouse colon at the dose of 120 mg/kg; ABX mice received an antibiotic cocktail (ampicillin, streptomycin and clindamycin 1 mg/mL each) starting 14 days before DNBS administration and for the whole length of the experiment. Mice were killed three days after DNBS administration. Data are expressed as mean ± SEM of pmol/mg of tissue from 6–8 mice for each experimental group. Data were statistically analyzed using two-way ANOVA followed by the Fisher’s LSD test. *p < 0.05, **p < 0.01, ***p < 0.001 and ****p < 0.0001

**Fig. 5:**
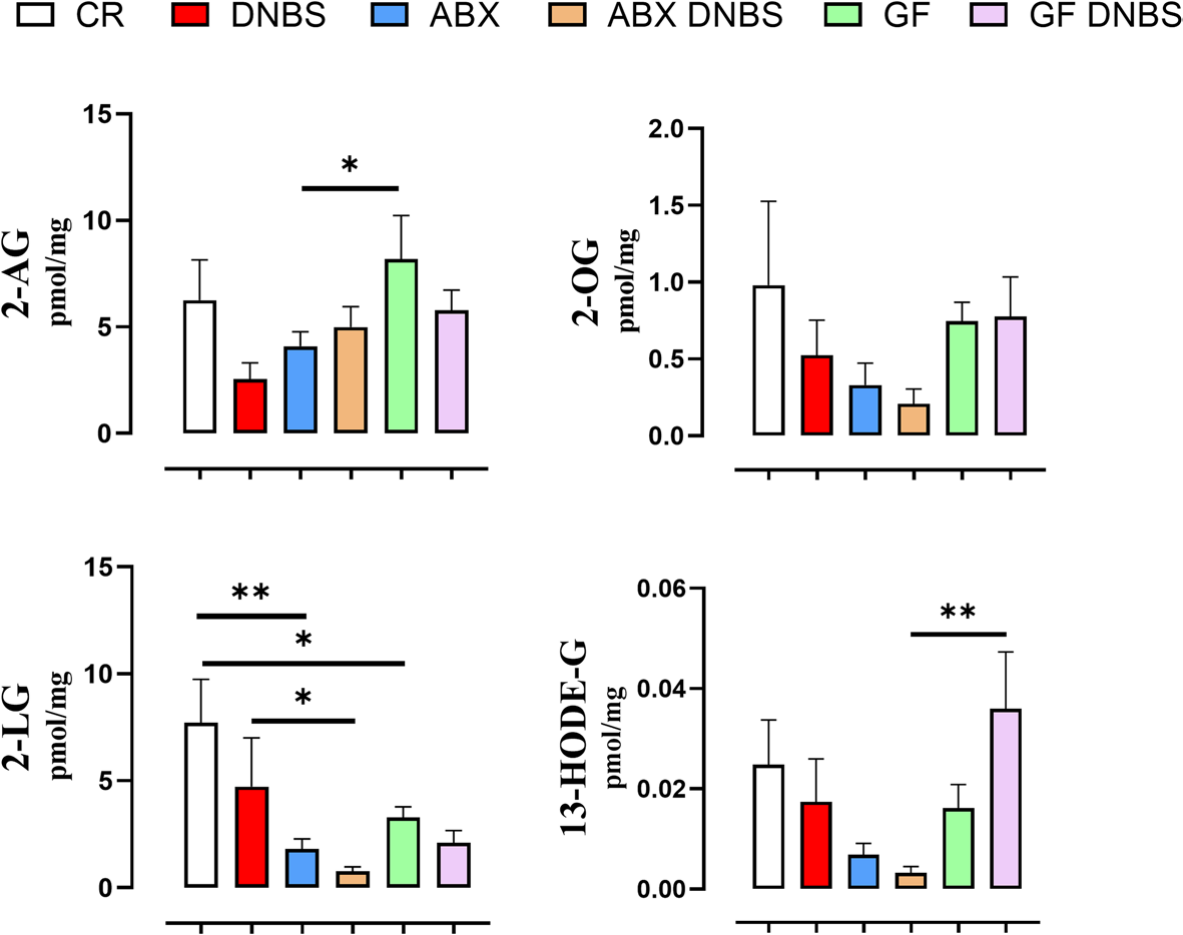
2-MAG, and 15-lipoxygenase 2-MAG derivative, levels in colon tissue from conventionally raised (CR), antibiotic-treated (ABX) and germ-free (GF) mice in healthy condition or under DNBS-induced inflammation. DNBS was rectally injected into the mouse colon at the dose of 120 mg/kg; ABX mice received an antibiotic cocktail (ampicillin, streptomycin and clindamycin 1 mg/mL each) starting 14 days before DNBS administration and for the whole length of the experiment. Mice were killed three days after DNBS administration. Data are expressed as mean ± SEM of pmol/mg of tissue from 6–8 mice for each experimental group. Data were statistically analyzed two-way ANOVA followed by the Fisher’s LSD test. *p < 0.05, **p < 0.01 and ***p < 0.001

To understand whether the difference in colon inflammatory response to DNBS in ABX and, especially, GF mice compared to CR mice is related to a different response with regard to the colon concentrations of eCBome mediators, we quantified these mediators in colonic tissue (Figs. 4 and 5).

Regarding NAEs levels, no differences were found in AEA and PEA basal levels between all groups (Fig. 4). In contrast, GF mice showed significantly higher basal levels of OEA, LEA and DHEA than CR and ABX mice; basal levels of 13-HODE-EA were also significantly higher in GF mice than in CR mice (Fig. 4). DNBS treatment had no significant effect on NAE levels in CR and ABX mice (Fig. 4). In contrast, the levels of AEA, OEA, LEA and DHEA were significantly reduced by DNBS administration in GF mice (Fig. 4). Yet, in inflamed tissues, the levels of LEA and DHEA were still significantly higher in GF than CR mice, and those of the LEA 15-lipoxygenase metabolite, 13-HODE-EA, was significantly higher in GF mice than both DNBS-treated CR and ABX mice (Fig. 4). In order to determine whether these alterations were due to changes in the expression of *Faah*, the main NAE metabolic enzyme, we went on to quantify *Faah* gene expression in our mice. While we found a trend for DNBS-induced decreases in *Faah* expression in general, this was only significant in the ABX mice, and no statistically significant differences among the various DNBS-treated cohorts were detected (Supp. Figure 1).

Regarding MAG levels, basal 2-AG levels were significantly higher in GF mice than in ABX mice (no difference was observed with CR mice) (Fig. 5). In contrast, lower basal 2-LG levels were observed in GF and ABX mice than in CR mice (Fig. 5). In all groups, DNBS administration had no significant effect on MAG levels (Fig. 5). In inflamed tissues, levels of 2-LG were significantly lower in ABX mice compared to CR, while levels of the 2-LG 15-lipoxygenase metabolite, 13-HODE-G, were significantly higher in GF than ABX mice (Fig. 5). No differences in 2-OG were detectable in mice under any condition.

Given that both AEA and 2-AG signal through the CB1 and CB2 receptors (encoded by *Cnr1* and *Cnr2*), which have previously been shown to play a role in the development of IBD [42, 43], we measured the gene expression of these two receptors in the colon. ABX mice tended to have increased *Cnr1* expression as compared to the other mice, an effect that was statistically significant only vs. ABX-DNBS mice (Supp. Figure 1), though, as for *Faah*, no statistically significant differences among the various DNBS-treated cohorts were detected. No differences in *Cnr2* expression were observed (Supp. Figure 1).

### Colonic endocannabinoidome bioactive lipid mediator levels negatively correlate with DNBS-induced inflammation

**Fig. 6:**
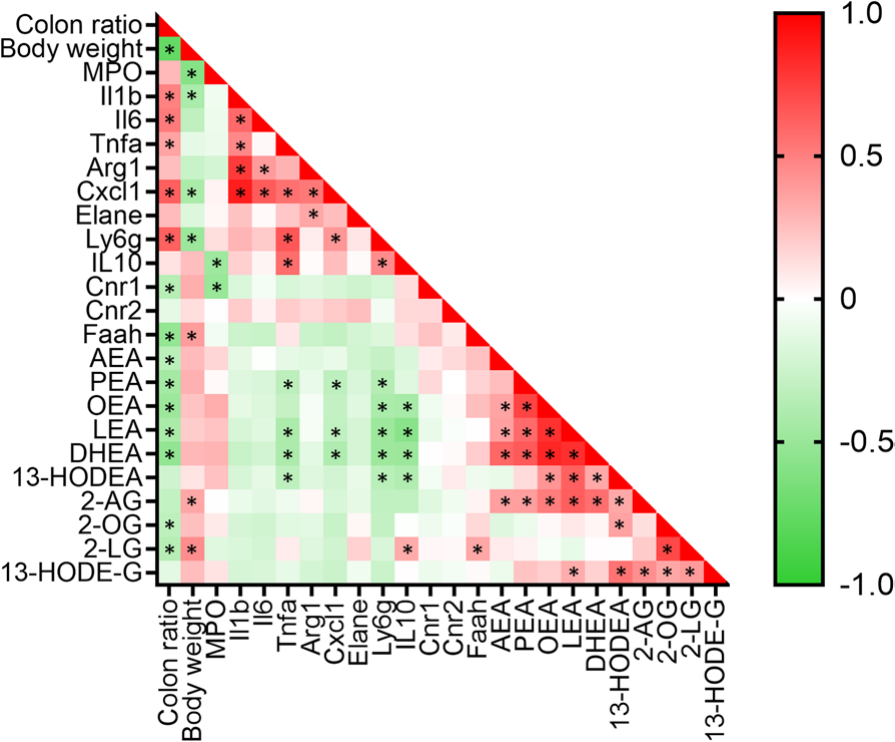
Correlation analysis between various parameters of colitis, inflammatory cytokine and neutrophil marker expression and eCBome gene expression and lipid mediator levels. Pearson’s r correlation coefficient is indicated from 1 (red, positively correlated) to -1 (green, negatively correlated). *p < 0.05

In order to gain an understanding of the relationship between the eCBome lipid mediator levels and the development of colitis and the inflammatory markers assessed above, we performed a correlation analysis (Fig. 6). As expected, body weight was negatively correlated while pro inflammatory cytokine gene expression levels (*Il1b*, *Il6* and *Tnfa*) and neutrophil markers (*Cxcl1* and *Ly6g*) were positively correlated with colon weight/length ratios in the mice. As would be expected, the neutrophil markers (especially *Cxcl1*) were positively correlated with the expression of inflammatory cytokines (*Il1b*, *Il6* and *Tnfa*). In marked contrast, *Cxcl1* and *Ly6g* were significantly negatively correlated with PEA, LEA and DHEA as well as OEA and 13-HODEA for *Ly6g*. In line with this, AEA, PEA, OEA, LEA, DHEA, 2-OG and 2-LG were all significantly negatively associated with colon weight/length ratio. While these lipid mediators were all generally also negatively correlated with the expression of inflammatory cytokines this was only statistically significant between PEA, LEA, DHEA or 13-HODEA and *Tnfa* expression. Taken together, these data show that potentially anti-inflammatory eCBome lipids, which are generally upregulated in GF mice, correlate negatively with macroscopic expression of inflammation (colon weight/length ratio) as well as with the expression of neutrophil markers and inflammatory cytokines.

## Discussion

IBD is a health problem that affect millions of people worldwide and for which there are no effective treatments. A plethora of recent studies have highlighted the involvement of gut dysbiosis (altered composition of the gut microbiota) in the development of this pathology [44]. The gut microbiota has also been shown to mediate the effects of pharmacological, nutraceutical and dietary interventions that have a positive impact on gut diseases [25, 26].

The eCBome has been described as an “expanded endocannabinoid system” and contains approximately 100 lipid mediators, 20 enzymes, and 20 receptors [10]. This complex system is one of the main targets responsible for the pharmacological effects of phytocannabinoids, active molecules found in the plant *Cannabis sativa* [10]. In addition, a close link between the eCBome and the gut microbiome (mBIome) has recently been established, known as the eCBome-mBIome axis [27, 28], which appears to play a role in several physiological and physio-pathological functions, including energy intake and processing, stress response, metabolism and autism spectrum disorders [29]. Moreover, the gut microbiota has been reported to influence, strongly and directly, eCBome signalling in the intestine and exploits eCBome signaling to exert some of its physio-pathological functions [28, 45]. In particular, Manca et al. showed that various small and large intestinal sections of GF and ABX mice exhibit higher levels of expression of CB1 and PPARα, two potentially beneficial receptors in colitis [46] and lower levels of GPR55 and GPR18, for which, instead a role in inflammation and intestinal immune response development, respectively, have been suggested [11, 47]. However, here, while CB1 mRNA tended to increase in the colons of ABX mice, we were unable to detect changes in GF mice, which may be due to the fact that we utilized Balb-c mice here compared to C57BL/6J mice in the work of Manca et al. Additionally, recent studies have shown that the anti-inflammatory effect of some molecules targeting the eCBome (CBD and CBDV) on colon inflammation in mice is linked with alteration of the gut microbiota composition [25, 26]. These observations may indicate that GF and ABX mice might respond differently to colitis-inducing stimuli, such as DNBS, which provided the rationale to carry out the present study. Indeed, in this work we have demonstrated that the existing link between eCBome and the gut microbiota (eCBome-mBIome axis) may impact on the development of colitis.

Using two experimental methods to affect the mouse gut microbiota (antibiotic cocktail and germ-free status), we showed that the impairment or absence of gut microbiota, especially in GF mice, reduces some aspects of inflammation in the DNBS-induced model of ulcerative colitis. GF mice exhibited decreased colon weight/length ratio and a delayed increase in the DAI score despite similar weight loss. Different inflammatory markers correlated significantly with eCBome lipid mediators that were altered within our experimental models suggesting that they may be partly the result of altered eCBome signalling. Indeed, GF mice exhibit elevated baseline levels of eCBome mediators with potentially anti-inflammatory effects (i.e. the NAEs OEA, LEA and DHEA, see below) [16, 21, 48]. These changes in eCBome signalling molecules do not appear to be linked to alterations in the expression of the main NAE degrading enzyme FAAH, .as previously reported in GF C57BL/6J mice [28], suggesting that the observed increases may be due to upregulation of biosynthetic pathways or increased levels of phospholipid precursors; possibilities that remain to be investigated.

In the DNBS-induced model, all groups treated with the pro-inflammatory agent developed intestinal inflammation, as shown by the macroscopic parameters of colon inflammation, i.e., colon weight/length ratio, body weight loss and DAI score. GF mice treated with DNBS showed lower levels of inflammation compared to mice from the CR and ABX groups, as the colon weight/ length ratio was significantly lower and the increase in DAI score was slower during the three days of observation. Furthermore, the kinetics of DAI development in GF mice differed markedly compared to CR and ABX mice, never reaching the peak observed at days 1 and 2 in DNBS-treated CR and ABX mice. While this is potentially a result of a muted inflammatory response in GF mice, we did not extend our studies out past day 3, and we cannot therefore be certain that the GF mice would not have a further increase in DAI or inflammatory parameters over time.

Looking at biochemical parameters of inflammation, DNBS treatment did not increase expression levels of pro-inflammatory cytokines in GF mice. Although not always in a significant manner, increases in *Il1b*, *Il6* and *Tnfa* expression levels were observed after DNBS treatment in CR and ABX mice. Similarly, MPO activity increased in DNBS-treated CR mice, but not in ABX and GF mice. Of note, basal levels of MPO activity were higher in GF mice than in CR and ABX mice, pointing to an altered intestinal immune response development in these mice. However, the basal expression levels of the major anti-inflammatory cytokine *Il10* were significantly lower in GF mice compared to CR and ABX mice; DNBS administration did not alter such levels in any group. Further, while the expression of neutrophil activation markers (*Cxcl1*, *Ly6g*, and *Arg1*) was not different in vehicle treated animals, GF mice did not show increases in the expression of these markers in a statistically significant manner, nor did they increase MPO activity. The expression of *Ly6g* was also blunted in DNBS-treated ABX animals, which also did not show significantly upregulated MPO activity in response to DNBS. These data suggest that antibiotics-treated and GF mice show altered DNBS-induced immune responses, as could be predicted by the well-known role of the gut microbiota in modulating immune defense under a plethora of conditions [49–51].

These results are partially consistent with the results of another study in which inflammation was induced in ABX-treated and GF mice with the phlogogen DSS [52]. The results of Hernández-Chirlaque et al. showed that GF mice developed minimal colonic inflammation, while ABX-treated mice developed milder inflammation compared to CR mice. More specifically, DSS-treated GF mice did not differ from CR mice in terms of weight loss and showed no increase in IL-1β levels and MPO activity (comparable to our results), but showed increased intestinal bleeding and mucosal barrier damage. However, in contrast to our results, the authors found similar basal MPO activity in GF and CR mice, whereas GF mice had increased levels of TNFα (after DNBS treatment), although with lower levels of IL-10, which instead is comparable to our results. Thus, the absence of microbiota appears in this previous study to have a dual effect on the response to DSS, i.e. attenuated inflammation but increased epithelial injury and bleeding, leading to worsening animal status. The differences between our and the study by Hernández-Chirlaque et al. could be ascribed to the different inflammatory agent, administration protocol and, particularly, mouse strain compared to our study. Nevertheless, it is interesting to note that despite the decrease in inflammation that we observed in GF mice (discussed below), these mice still lost comparable amounts of weight to CR controls; this may be due to, among other things, increased intestinal barrier damage, a possibility which remains to be investigated.

A recent study examining the role of the gut microbiome in mediating the protective effects of helminth infection on DNBS-induced colitis also utilized both antibiotics-treated and GF mice [53]. While no direct comparison of the development of colitis was performed between these models, the authors did report a significant increase in DAI at day 3, and did not observe in increase in IL-10 levels, which is consistent with what we now report. Unfortunately, only an endpoint analysis was presented, and thus it is unclear if the progression of DAI development in GF animals was different from conventionally raised or antibiotics-treated mice. The lipidomics analysis of the mouse colon revealed that eCBome lipid mediators were present in different concentrations depending on the status of the gut microbiota and responded differently to colon inflammation in GF mice with respect to CR and ABX-treated mice. At the basal level, i.e., in non-inflamed mice, and partly in agreement with previous findings [28], GF mice exhibited significantly higher levels of anti-inflammatory NAEs such as OEA, LEA, DHEA and 13-hydroxy-octadecadienoicethanolamide (13-HODE-EA), which act at different receptors (PPARα, TRPV1 and TRPV2 [54]), compared to CR and ABX-treated healthy mice; a trend towards an increase was also seen in AEA levels, but this was not statistically significant. It is important to note that while we have utilized an antibiotics regimen previously shown to effectively reduce the overall bacterial levels within the intestines of mice [33], this will not result in the elimination of all bacteria, not to mention the persistence of other microorganisms (e.g. archaea, fungi, and viruses), which are also able to affect the host and are lacking in the GF mice. Furthermore, ABX-treated mice will not be subject to the developmental alterations in the gut and immune system that are well documented in GF mice [49–51]. We suspect that these differences may well impact the gut eCBome in unique ways. Additionally, we have shown that reconstitution of the gut microbiome in GF mice rescues only a subset of eCBome lipid and gene expression levels within the intestines [28], indicating that some of these changes are not limited to direct modification by microorganisms but are also a consequence of altered gut development in GF mice.

OEA has been reported to exert anti-inflammatory effects in experimental colitis [16], while LEA is able to inhibit NF-kB signalling and thus exert anti-inflammatory effects [48]. DHEA, synthesised by NAPE-PLD from the n-3 PUFA DHA, has been reported to have a strong anti-inflammatory effect, even stronger than AEA [21]. Thus, higher levels of anti-inflammatory mediators in GF mice could at least partly explain the lower propension to develop inflammation and the lower production of pro-inflammatory cytokines following DNBS, and this effect could be explained as a compensatory effect of the eCBome due to the absence of the gut microbiota [28]. It is also interesting to note that, in CR Balb-c mice, we could not observe here the increases in AEA, 2-AG or PEA colonic levels previously reported in ICR mice following DNBS [22], thus suggesting the existence of mouse strain-specific effects of DNBS on the intestinal eCBome.

The 13-HODE-EA and 13-HODE-G are produced in human neutrophils and eosinophils from the 15-lipoxygenase (15-LOX)-catalysed oxidation of LEA and 2-LG, respectively, but the biological activity of these mediators is still unclear, although an anti-inflammatory role has again been suggested [7, 8]. Interestingly, DNBS-treated GF mice exhibited higher concentrations of these LEA and 2-LG metabolites, and this increase was accompanied by the reduction of LEA and 2-LG in DNBS-treated mice compared to healthy GF mice, suggesting that high concentrations of 15-LOX metabolites (13-HODE) are produced in the inflammatory process as consequence of a higher concentration of the precursors under healthy conditions, consequently resulting in a decrease in the level of these same precursors. Given that n-3 FA-derived 15-LOX metabolites have been shown to protect against DSS-induced inflammation [55], it is interesting to speculate that in GF mice increased 13-HODE-EA may have a similar effect. However, further studies are needed to unravel the biological significance of these differences, especially with regard to the altered levels of the newly identified 13-HODE derivatives.

While our data (and those of many others) suggest that microbiome-targeting/antibiotic treatments may be an effective therapeutic for the treatment of IBDs, the clinical benefits of this, and for those patients specifically, is still very much an open question [56, 57]. The work presented here suggests that microbiome-mediated effects on the eCBome, which can then interact with the immune system, may contribute to the mechanism through which such treatments may elicit their effects, as demonstrated by the significant negative correlation between eCBome lipid mediator levels and measures of colitis, inflammation and neutrophil markers, while these latter generally correlate positively with colitis features and the expression of inflammatory cytokines in the colon. Future pharmacological studies in which colitis models are treated with these eCBome mediators and/or pharmacological tools manipulating their levels or several potential receptors, will be of the utmost interest to support their therapeutic potential for colitis/IBD patients.

In summary, we have shown here that mice raised in GF conditions develop a lower degree of inflammation in the experimental model of DNBS-induced ulcerative colitis, whereas mice with a depletion of the gut microbiota achieved by ABX treatment are mostly devoid of such property. The complete and congenital lack of gut microbiota and the resulting differential development of the gut immune system in GF mice appears to be followed by a potentially compensatory effect on mediators of the eCBome, which may help explain the lower susceptibility of these mice to develop ulcerative colitis and its inflammatory markers. In a nutshell, we suggest that the relationship between the gut microbiota and the eCBome affects the development of colon inflammation.

## Supplementary Information

**Additional file 1: Supplementary Figure 1.** Gene expressions of receptors (*Cnr1*, *Cnr2*) and enzyme (*Faah*) belonging to eCBome in colon tissue from conventionally raised (CR), antibiotic- treated (ABX) and germ-free (GF) mice in healthy condition or under DNBS-induced inflammation. DNBS was rectally injected into the mouse colon at the dose of 120 mg/kg; ABX mice received an antibiotic cocktail (ampicillin, streptomycin and clindamycin 1 mg/mL each) starting 14 days before DNBS administration and for the whole length of the experiment. Mice were killed three days after DNBS administration. Data are expressed as mean ± SEM of 6-8 mice for each experimental group. Data were statistically analyzed using two-way ANOVA followed by the Fisher’s LSD test. *p<0.05, **p<0.01

## Data Availability

Not applicable.
