# Supplementary figures and images for: Altered endocannabinoidome bioactive lipid levels accompany reduced DNBS-induced colonic inflammation in germ-free mice
Source: Lipids Health Dis. 2023 May 15;22:63. doi: 10.1186/s12944-023-01823-1 (PMC10186685; doi:10.1186/s12944-023-01823-1)

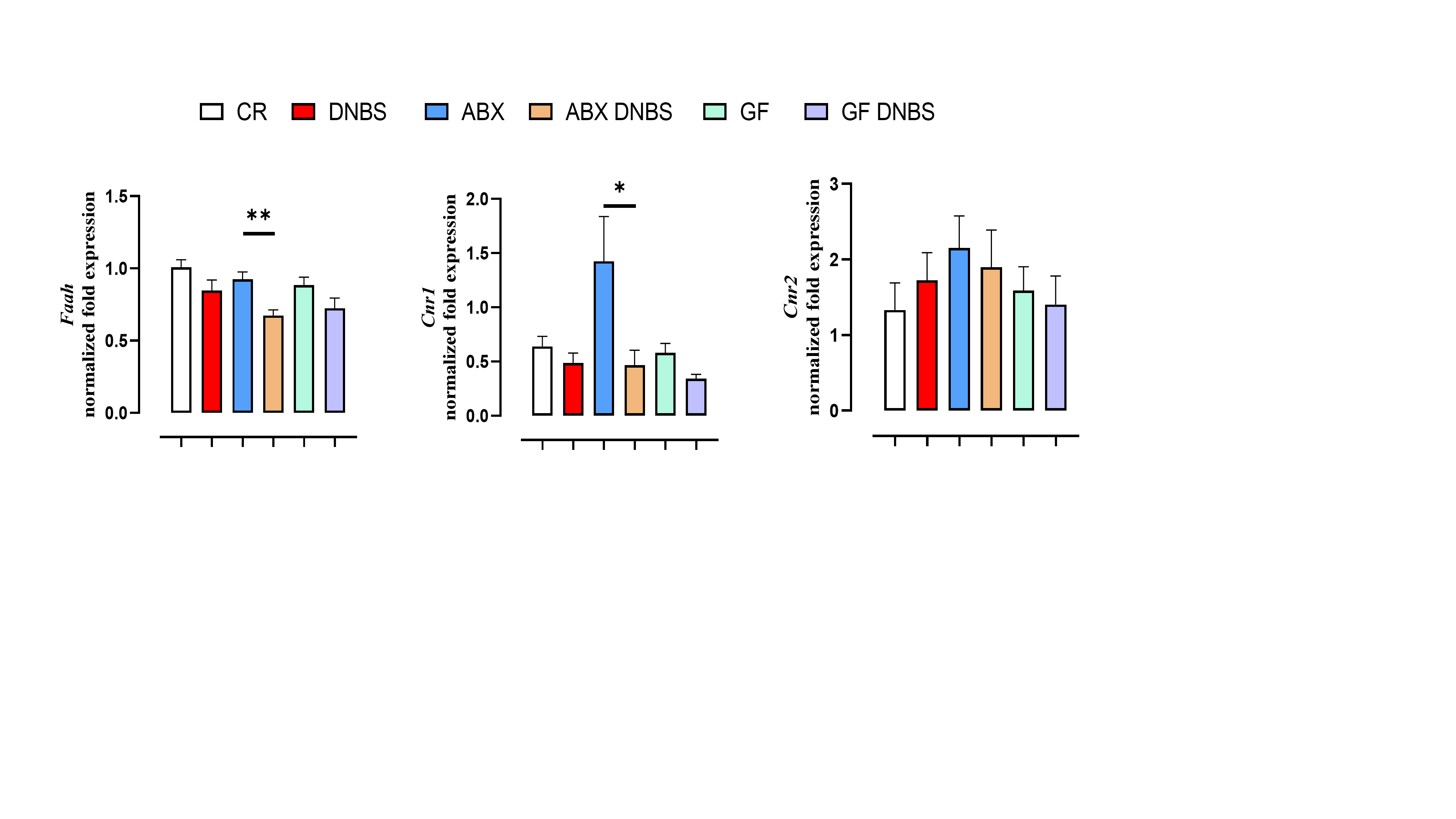

Supplement: Supplementary file 1 — Additional file 1: Supplementary Figure 1. Gene expressions of receptors (Cnr1, Cnr2) and enzyme (Faah) belonging to eCBome in colon tissue from conventionally raised (CR), antibiotic- treated (ABX) and germ-free (GF) mice in healthy condition or under DNBS-induced inflammation. DNBS was rectally injected into the mouse colon at the dose of 120 mg/kg; ABX mice received an antibiotic cocktail (ampicillin, streptomycin and clindamycin 1 mg/mL each) starting 14 days before DNBS administration and for the whole length of the experiment. Mice were killed three days after DNBS administration. Data are expressed as mean ± SEM of 6-8 mice for each experimental group. Data were statistically analyzed using two-way ANOVA followed by the Fisher’s LSD test. *p<0.05, **p<0.01 [file 12944_2023_1823_MOESM1_ESM.tif]
